# Supplementary material for: Continuous in situ synthesis of a complete set of tRNAs sustains steady-state translation in a recombinant cell-free system
Source: Nat Commun. 2025 Jul 5;16:6212. doi: 10.1038/s41467-025-61671-8 (PMC12228740; doi:10.1038/s41467-025-61671-8)
Supplement: Supplementary file 1 — Supplementary Information [file 41467_2025_61671_MOESM1_ESM.pdf]

Continuous *in situ* synthesis of a complete set of tRNAs sustains steady-state translation in a recombinant cell-free system.

Fanjun Li †1, Amogh Kumar Baranwal †1, and Sebastian J. Maerkl*1

[1] Institute of Bioengineering, School of Engineering, École Polytechnique Fédérale de Lausanne

June 18, 2025

†These authors contributed equally to this work

*Correspondence: sebastian.maerkl@epfl.ch

Supporting Information

Supplementary Figures S1 to S11

Supplementary Tables S1 to S6

Supplementary Figures and Tables

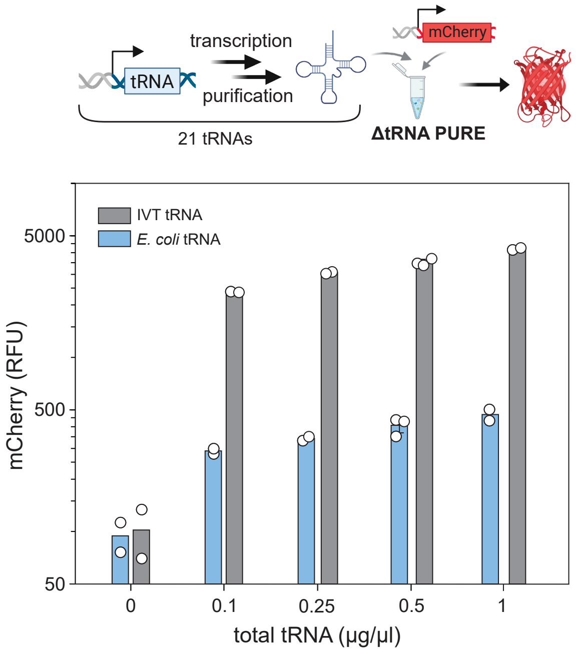

Supplementary Figure 1: **mCherry synthesis with IVT tRNAs.** A schematic of mCherry synthesis with IVT tRNAs in ΔtRNA PURE system (top). The schematic was partially generated using BioRender. In all PURE reactions, 4 nM mCherry template was used with indicated amount of tRNA. The fluorescence of mCherry with indicated tRNA in ΔtRNA PURE system was shown (bottom) (n = 2 independent experiments for 0, 0.1, 0.25 and 1 μg/μl IVT tRNAs and *E.coli* tRNA, n = 3 for 0.5 μg/μl IVT tRNAs and *E.coli* tRNA). Bars and error bars represent the mean and standard deviation. Source data are provided as a Source Data file.

T7 promotor: green tRNA gene: blue Nt.BspQI site: red T7 terminator: pink

tcgcgcgtttcggtgatgacggtgaaaacctctgacacatgcagctcccggagacggtcacagcttgtctgtaagcggatgccgggagcagacaagcccgtcagggcgcgtcagcgggtgttggcgggtgtcggggctggctt
aactatgcggcatcagagcagattgtactgagagtgcaccatatgcggtgtgaaataccgcacagatgcgtaaggagaaaataccgcatcaggcgccattcgccattcaggctgcgcaactgttgggaagggcgatcggtgc
gggcctcttcgctattacgccagctggcgaaagggggatgtgctgcaaggcgattaagttgggtaacgccagggttttcccagtcacgacgttgtaaaacgacggccagtgaattcgagctcggtacccccgcgtaatacgac
tcactataggggctatagctcagctgggagagcgcttgcatggcatgcaagaggtcagcggttcgatcccgcttagctccaccaagaagagcccgcgtaatacgactcactatagcgcccgtagctcagctggat
agagcgctgccctccggaggcagaggtctcaggttcgaatcctgtcgggcgcgccaagaagagcccgcgtaatacgactcactatagcctctgtagttcagtcggtagaacggcggactgttaatccgtatgtca
ctggttcgagtccagtcagaggcgccaagaagagcccgcgtaatacgactcactataggagcggtagttcagtcggttagaatacctgcctgtcacgcagggggtcgcgggttcgagtcccgtccgttccgccaa
gaagagcccgcgtaatacgactcactataggcgcgttaacaaagcggttatgtagcggattgcaaatccgtctagtccggttcgactccggaacgcgcctccaagaagagcccgcgtaatacgactcactatag
gcggggtggagcagcctggtagctcgtcgggctcataacccgaagatcgtcggttcaaatccggcccccgcaaccaagaagagcccgcgtaatacgactcactatatggggtatcgccaagcggtaaggcacc
ggattctgattccggcattccgaggttcgaatcctcgtaccccagccaagaagagcccgcgtaatacgactcactatagtccccttcgtctagaggcccaggacaccgccctctcacggcggtaacaggggttcg
aatcccctaggggacgccaagaagagcccgcgtaatacgactcactatagcgggaatagctcagttggtagagcacgaccttgccaaggtcggggtcgcgagttcgagtctcgtttcccgctccaagaagagcc
cgcgtaatacgactcactataggtggctatagctcagttggtagagccctggattgtgattccagttgtcgtgggttcgaatcccattagccaccccaagaagagcccgcgtaatacgactcactattaggcttgtag
ctcaggtggttagagcgcacccctgataagggtgaggtcggtggttcaagtccactcaggcctaccaagaagagcccgcgtaatacgactcactatagcgaaggtggcggaattggtagacgcgctagcttcagg
tgttagtgtccttacggacgtgggggttcaagtccccccctcgcaccaagaagagcccgcgtaatacgactcactatagggtcgttagctcagttggtagagcagttgactcttaatcaattggtcgcaggttcga
atcctgcacgacccaccaagaagagcccgcgtaatacgactcactataggctacgtagctcagttggttagagcacatcactcataatgatggggtcacaggttcgaatcccgtcgtagccaccaagaagagcc
cgcgtaatacgactcactatagcccggatagctcagtcggtagagcaggggattgaaaatccccgtgtccttggttcgattccgagtccgggcaccaagaagagcccgcgtaatacgactcactatacggcacgt
agcgcagcctggtagcgcaccgtcatggggtgtcgggggtcggaggttcaaatcctctcgtgccgaccaagaagagcccgcgtaatacgactcactataggtgaggtgtccgagtggctgaaggagcacgcctg
gaaagtgtgtatacggcaacgtatcgggggttcgaatccccccctcaccgccaagaagagcccgcgtaatacgactcactatagctgatatggctcagttggtagagcgcacccttggtaagggtgaggtcccca
gttcgactctgggtatcagcaccaagaagagcccgcgtaatacgactcactattaggggcgtagttcaattggtagagcaccggtctccaaaaccgggtgttgggagttcgagtctctccgcccctgccaagaaga
gcccgcgtaatacgactcactataggtggggttcccgagcggccaaagggagcagactgtaaatctgccgtcacagacttcgaaggttcgaatccttcccccaccaccaagaagagcccgcgtaatacgactc
actatagcgtccgtagctcagttggttagagcaccaccttgacatggtgggggtcggtggttcgagtccactcggacgcaccaagaagagcaaccccttggggcctctaaacgggtcttgaggggttttttttgggga
tcctctagagtcgacctgcaggcatgcaagcttggcgtaatcatggtcatagctgtttcctgtgtgaaattgttatccgctcacaattccacacaacatacgagccggaagcataaagtgtaaagcctggggtgcctaatgagtg
agctaactcacattaattgcgttgcgctcactgcccgctttccagtcgggaaacctgtcgtgccagctgcattaatgaatcggccaacgcgcggggagaggcggtttgcgtattgggcgctcttccgcttcctcgctcactgactc
gctgcgctcggtcgttcggctgcggcgagcggtatcagctcactcaaaggcggtaatacggttatccacagaatcaggggataacgcaggaaagaacatgtgagcaaaaggccagcaaaaggccaggaaccgtaaaaag
gccgcgttgctggcgtttttccataggctccgcccccctgacgagcatcacaaaaatcgacgctcaagtcagaggtggcgaaacccgacaggactataaagataccaggcgtttccccctggaagctccctcgtgcgctctc
ctgttccgaccctgccgcttaccggatacctgtccgcctttctcccttcgggaagcgtggcgctttctcatagctcacgctgtaggtatctcagttcggtgtaggtcgttcgctccaagctgggctgtgtgcacgaaccccccgttc
agcccgaccgctgcgccttatccggtaactatcgtcttgagtccaacccggtaagacacgacttatcgccactggcagcagccactggtaacaggattagcagagcgaggtatgtaggcggtgctacagagttcttgaagtgg
tggcctaactacggctacactagaaggacagtatttggtatctgcgctctgctgaagccagttaccttcggaaaaagagttggtagctcttgatccggcaaacaaaccaccgctggtagcggtggtttttttgtttgcaagcagca
gattacgcgcagaaaaaaaggatctcaagaagatcctttgatcttttctacggggtctgacgctcagtggaacgaaaactcacgttaagggattttggtcatgagattatcaaaaaggatcttcacctagatccttttaaattaaa
aatgaagttttaaatcaatctaaagtatatatgagtaaacttggtctgacagttaccaatgcttaatcagtgaggcacctatctcagcgatctgtctatttcgttcatccatagttgcctgactccccgtcgtgtagataactacgata
cgggagggcttaccatctggccccagtgctgcaatgataccgcgagacccacgctcaccggctccagatttatcagcaataaaccagccagccggaagggccgagcgcagaagtggtcctgcaactttatccgcctccat
ccagtctattaattgttgccgggaagctagagtaagtagttcgccagttaatagtttgcgcaacgttgttgccattgctacaggcatcgtggtgtcacgctcgtcgtttggtatggcttcattcagctccggttcccaacgatcaagg
cgagttacatgatcccccatgttgtgcaaaaaagcggttagctccttcggtcctccgatcgttgtcagaagtaagttggccgcagtgttatcactcatggttatggcagcactgcataattctcttactgtcatgccatccgtaagat
gcttttctgtgactggtgagtactcaaccaagtcattctgagaatagtgtatgcggcgaccgagttgctcttgcccggcgtcaatacgggataataccgcgccacatagcagaactttaaaagtgctcatcattggaaaacgttct
tcggggcgaaaactctcaaggatcttaccgctgttgagatccagttcgatgtaacccactcgtgcacccaactgatcttcagcatcttttactttcaccagcgtttctgggtgagcaaaaacaggaaggcaaaatgccgcaaaa
aagggaataagggcgacacggaaatgttgaatactcatactcttcctttttcaatattattgaagcatttatcagggttattgtctcatgagcggatacatatttgaatgtatttagaaaaataaacaaataggggttccgcgcacatt
tccccgaaaagtgccacctgacgtctaagaaaccattattatcatgacattaacctataaaaataggcgtatcacgaggccctttcgtc

Supplementary Figure 2: **DNA sequence of pUC19_21 tRNA genes (4991 bp).** A full sequence information of pUC19 containing 21 tRNA genes. The sequence of T7 promotors, tRNA genes, NtBspQI sites and T7 terminators are colored in green, blue, red and pink.

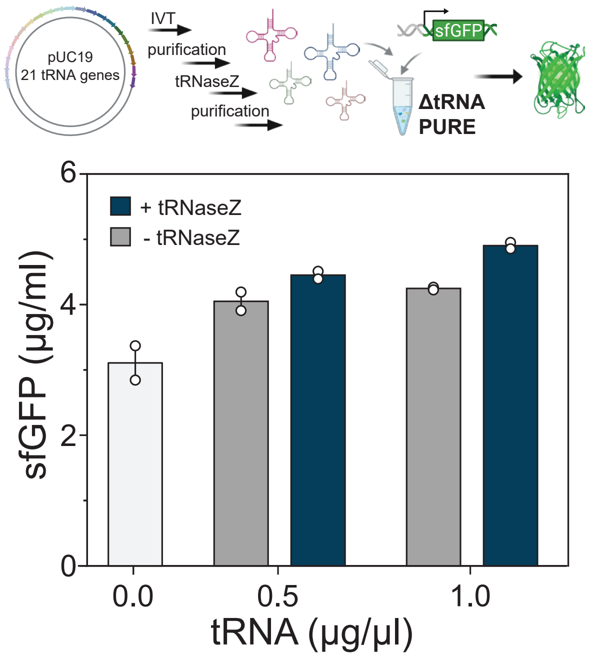

Supplementary Figure 3: **sfGFP synthesis with commercial energy solution and tRNase Z treated tRNAs**. The schematic above the plot was partially created with BioRender. In all PURE reactions, 4 nM sfGFP template was used with indicated amount of tRNA. The yield of sfGFP with indicated tRNA in ΔtRNA PURE system was shown (bottom) (n = 2 independent experiments). Each dot represents one replicated. Bars represent the mean. Source data are provided as a Source Data file.

gcgcaacgcaattaatgtaagttagctcactcattaggcaccgggatctcgaccgatgcccttgagagccttcaacccagtcagctccttccggtgggcgcggggcatgactatcgtcgccgcacttatgactgtgttctttat
catgcaactcgtaggacaggtgccggcagcgctctgggtcattttcggcgaggaccgctttcgctggagcgcgacgatgatcggcctgtcgcttgcggtattcggaatcttgcacgccctcgctcaagccttcgtcactggtcc
cgccaccaaacgtttcggcgagaagcaggccattatcgccggcatggcggccccacgggtgcgcatgatcgtgctcctgtcgttgaggacccggctaggctggcggggttgccttactggttagcagaatgaatcaccgat
acgcgagcgaacgtgaagcgactgctgctgcaaaacgtctgcgacctgagcaacaacatgaatggtctttggtttccgtgtttcgtaaagtctggaaacgcggaagtcagcgccctgcaccattatgttccggatctgcatcg
caggatgctgctggctaccctgtggaacacctacatctgtattaacgaagcgctggcattgaccctgagtgatttttctctggtcccgccgcatccataccgccagttgtttaccctcacaacgttccagtaaccgggcatgtt
catcatcagtaacccgtatcgtgagcatcctctctcgtttcatcggtatcattacccccatgaacagaaatcccccttacacggaggcatcagtgaccaaacaggaaaaaaccgcccttaacatggcccgctttatcagaa
gccagacattaacgcttctggagaaactcaacgagctggacgcggatgaacaggcagacatctgtgaatcgcttcacgaccacgctgatgagctttaccgcagctgcctcgcgcgtttcggtgatgacggtgaaaacctct
gacacatgcagctcccggtgacggtcacagcttgtctgtaagcggatgccgggagcagacaagcccgtcagggcgcgtcagcgggtgttggcgggtgtcggggcgcagccatgacccagtcacgtagcgatagcggagt
gtatactggcttaactatgcggcatcagagcagattgtactgagagtgcaccatatatgcggtgtgaaataccgcacagatgcgtaaggagaaaataccgcatcaggcgctcttccgcttcctcgctcactgactcgctgcgc
tcggtcgttcggctgcggcgagcggtatcagctcactcaaaggcggtaatacggttatccacagaatcaggggataacgcaggaaagaacatgtgagcaaaaggccagcaaaaggccaggaaccgtaaaaaggccgcg
ttgctggcgtttttccataggctccgcccccctgacgagcatcacaaaaatcgacgctcaagtcagaggtggcgaaacccgacaggactataaagataccaggcgtttccccctggaagctccctcgtgcgctctcctgtt
ccgaccctgccgcttaccggatacctgtccgcctttctcccttcgggaagcgtggcgctttctcatagctcacgctgtaggtatctcagttcggtgtaggtcgttcgctccaagctgggctgtgtgcacgaaccccccgttcag
cccgaccgctgcgccttatccggtaactatcgtcttgagtccaacccggtaagacacgacttatcgccactggcagcagccactggtaacaggattagcagagcgaggtatgtaggcggtgctacagagttcttgaagtggt
ggcctaactacggctacactagaaggacagtatttggtatctgcgctctgctgaagccagttaccttcggaaaaagagttggtagctcttgatccggcaaacaaaccaccgctggtagcggtggtttttttgtttgcaagcagc
agattacgcgcagaaaaaaaggatctcaagaagatcctttgatcttttctacggggtctgacgctcagtggaacgaaaactcacgttaagggattttggtcatgagattatcaaaaaggatcttcacctagatccttttaaatta
aaaatgaagttttaaatcaatctaaagtatatatgagtaaacttggtctgacagttaccaatgcttaatcagtgaggcacctatctcagcgatctgtctatttcgttcatccatagttgcctgactccccgtcgtgtagataactac
gatacgggagggcttaccatctggccccagtgctgcaatgataccgcgagagccacgctcaccggctccagatttatcagcaataaaccagccagccggaagggccgagcgcagaagtggtcctgcaactttatccgcc
tccatccagtctattaattgttgccgggaagctagagtaagtagttcgccagttaatagtttgcgcaacgttgttgccattgctacaggcatcgtggtgtcacgctcgtcgtttggtatggcttcattcagctccggttcccaacgat
caaggcgagttacatgatcccccatgttgtgcaaaaaagcggttagctccttcggtcctccgatcgttgtcagaagtaagttggccgcagtgttatcactcatggttatggcagcactgcataattctcttactgtcatgccatcc
gtaagatgcttttctgtgactggtgagtactcaaccaagtcattctgagaatagtgtatgcggcgaccgagttgctcttgcccggcgtcaatacgggataataccgcgccacatagcagaactttaaaagtgctcatcattgga
aaacgttcttcggggcgaaaactctcaaggatcttaccgctgttgagatccagttcgatgtaacccactcgtgcacccaactgatcttcagcatcttttactttcaccagcgtttctgggtgagcaaaaacaggaaggcaaaa
tgccgcaaaaaagggaataagggcgacacggaaatgttgaatactcatactcttcctttttcaatattattgaagcatttatcagggttattgtctcatgagcggatacatatttgaatgtatttagaaaaataaacaaataggggt
tccgcgcctaggctagcataaccccttggggcctctaaacgggtcttgaggggttttttgcaaataaaacgaaaggctcagtcgaaagactgggcctttcgttttatctgttgtttgtcggtgaacgctctcctgagtaggacaaa
tccgccgggagcggatttgaacgttgcgaagcaacggcccggagggtggcgggcaggacgcccgccataaactgccaggcatcaaattaagcagaaggccatcctgacggatggcctttttgcgtttctacaaactctctg
gccctagagaacccactgcttacagttgggtaacgccagggttttcccagtcacgacgttgtaaaacgacggccagtccgcgtaatacgactcactataggggctatagctcagctgggagagcgcttgcatggcatgcaa
gaggtcagcggttcgatcccgcttagctccaccaagaagagcaacccccttggggcctctaaacgggtcttgaggggttttttttccgcgtaatacgactcactatagcgcccgtagctcagctggatagagcgctgccctccg
gaggcagaggtctcaggttcgaatcctgtcgggcgcgccaagaagagcaacccccttggggcctctaaacgggtcttgaggggttttttttccgcgtaatacgactcactatagcctctgtagttcagtcggtagaacggcgga
ctgttaatccgtatgtcactggttcgagtccagtcagaggcgccaagaagagcaacccccttggggcctctaaacgggtcttgaggggttttttttccgcgtaatacgactcactataggagcggtagttcagtcggttagaatac
ctgcctgtcacgcagggggtcgcgggttcgagtcccgtccgttccgccaagaagagcaacccccttggggcctctaaacgggtcttgaggggttttttttccgcgtaatacgactcactataggcgcgttaacaaagcggttatg
tagcggattgcaaatccgtctagtccggttcgactccggaacgcgcctccaagaagagcaacccccttggggcctctaaacgggtcttgaggggttttttttccgcgtaatacgactcactataggcggggtggagcagcctgg
tagctcgtcgggctcataacccgaagatcgtcggttcaaatccggcccccgcaaccaagaagagcaacccccttggggcctctaaacgggtcttgaggggttttttttccgcgtaatacgactcactatatggggtatcgcca
agcggtaaggcaccggattctgattccggcattccgaggttcgaatcctcgtaccccagccaagaagagcaacccccttggggcctctaaacgggtcttgaggggttttttttccgcgtaatacgactcactatagtccccttcg
tctagaggcccaggacaccgccctctcacggcggtaacaggggttcgaatcccctaggggacgccaagaagagcaacccccttggggcctctaaacgggtcttgaggggttttttttccgcgtaatacgactcactatagcg
ggaatagctcagttggtagagcacgaccttgccaaggtcggggtcgcgagttcgagtctcgtttcccgctccaagaagagcaacccccttggggcctctaaacgggtcttgaggggttttttttccgcgtaatacgactcactat
aggtggctatagctcagttggtagagccctggattgtgattccagttgtcgtgggttcgaatcccattagccaccccaagaagagcaacccccttggggcctctaaacgggtcttgaggggttttttttccgcgtaatacgactca
ctattaggcttgtagctcaggtggttagagcgcacccctgataagggtgaggtcggtggttcaagtccactcaggcctaccaagaagagcaacccccttggggcctctaaacgggtcttgaggggttttttttccgcgtaatacg
actcactatagcgaaggtggcggaattggtagacgcgctagcttcaggtgttagtgtccttacggacgtgggggttcaagtccccccccctcgcaccaagaagagcaacccccttggggcctctaaacgggtcttgaggggtttt
ttttccgcgtaatacgactcactatagggtcgttagctcagttggtagagcagttgactcttaatcaattggtcgcaggttcgaatcctgcacgacccaccaagaagagcaacccccttggggcctctaaacgggtcttgagggg
ttttttttccgcgtaatacgactcactataggctacgtagctcagttggttagagcacatcactcataatgatggggtcacaggttcgaatcccgtcgtagccaccaagaagagcaacccccttggggcctctaaacgggtcttg
aggggttttttttccgcgtaatacgactcactatagcccggatagctcagtcggtagagcaggggattgaaaatccccgtgtccttggttcgattccgagtccgggcaccaagaagagcaacccccttggggcctctaaacggg
tcttgaggggttttttttccgcgtaatacgactcactatacggcacgtagcgcagcctggtagcgcaccgtcatggggtgtcgggggtcggaggttcaaatcctctcgtgccgaccaagaagagcaacccccttggggcctcta
aacgggtcttgaggggttttttttccgcgtaatacgactcactataggtgaggtgtccgagtggctgaaggagcacgcctggaaagtgtgtatacggcaacgtatcgggggttcgaatcccccccctcaccgccaagaagagc
aacccccttggggcctctaaacgggtcttgaggggttttttttccgcgtaatacgactcactatagctgatatggctcagttggtagagcgcacccttggtaagggtgaggtccccagttcgactctgggtatcagcaccaagaag
agcaacccccttggggcctctaaacgggtcttgaggggttttttttccgcgtaatacgactcactataaggggcgtagttcaattggtagagcaccggtctccaaaaccgggtgttgggagttcgagtctctccgcccctgccaa
gaagagcaacccccttggggcctctaaacgggtcttgaggggttttttttccgcgtaatacgactcactataggtggggttcccgagcggccaaagggagcagactgtaaatctgccgtcacagacttcgaaggttcgaatcct
tcccccaccaccaagaagagcaacccccttggggcctctaaacgggtcttgaggggttttttttccgcgtaatacgactcactatagcgtccgtagctcagttggttagagcaccaccttgacatggtgggggtcggtggttcga
gtccactcggacgcaccaagaagagcaacccccttggggcctctaaacgggtcttgaggggttttttttggcgtaatcatggtcatagctgtttcctgtgtgaaattgttatccgctcacaattccacacaagaaggtgccactcc
cactgttttgtagaaacgcaaaaaggccatccgtcaggatggccttctgcttaatttgatgcctggcagtttatggcgggcgtcctgcccgccaccctccgggccgttgcttcgcaacgttcaaatccgctcccggcggatttgt
cctactcaggagagcgttcaccgacaaacaacagataaaacgaaaggcccagtctttcgactgagcctttcgttttatttgtttacactttatgcttccggctcgtatgttgcctctagaaataattttgtttaactttaagaagga
gatatacatatg

Supplementary Figure 4: DNA sequence of pET-A_21 tRNA genes (7038 bp).

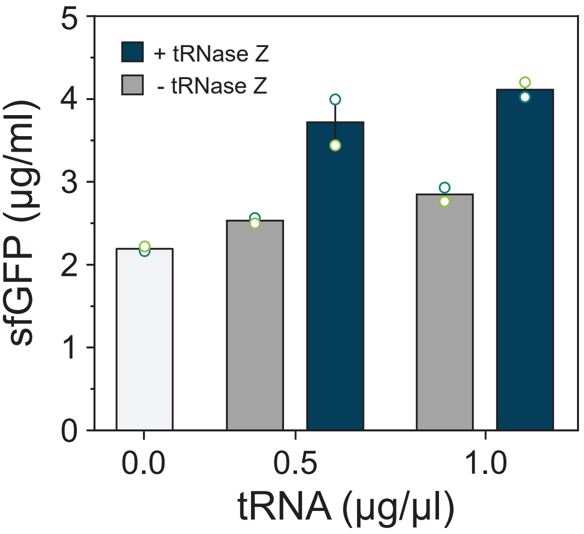

Supplementary Figure 5: **sfGFP synthesis with pre-tRNAs from plasmid template pET-A_21 tRNAs**. In all PURE reactions, 4 nM sfGFP template was used with indicated amount of tRNA. The yield of sfGFP with indicated tRNA in ΔtRNA PURE system was shown (n = 2 independent experiments). Each dot represents one replicated. Bars represent the mean. Source data are provided as a Source Data file.

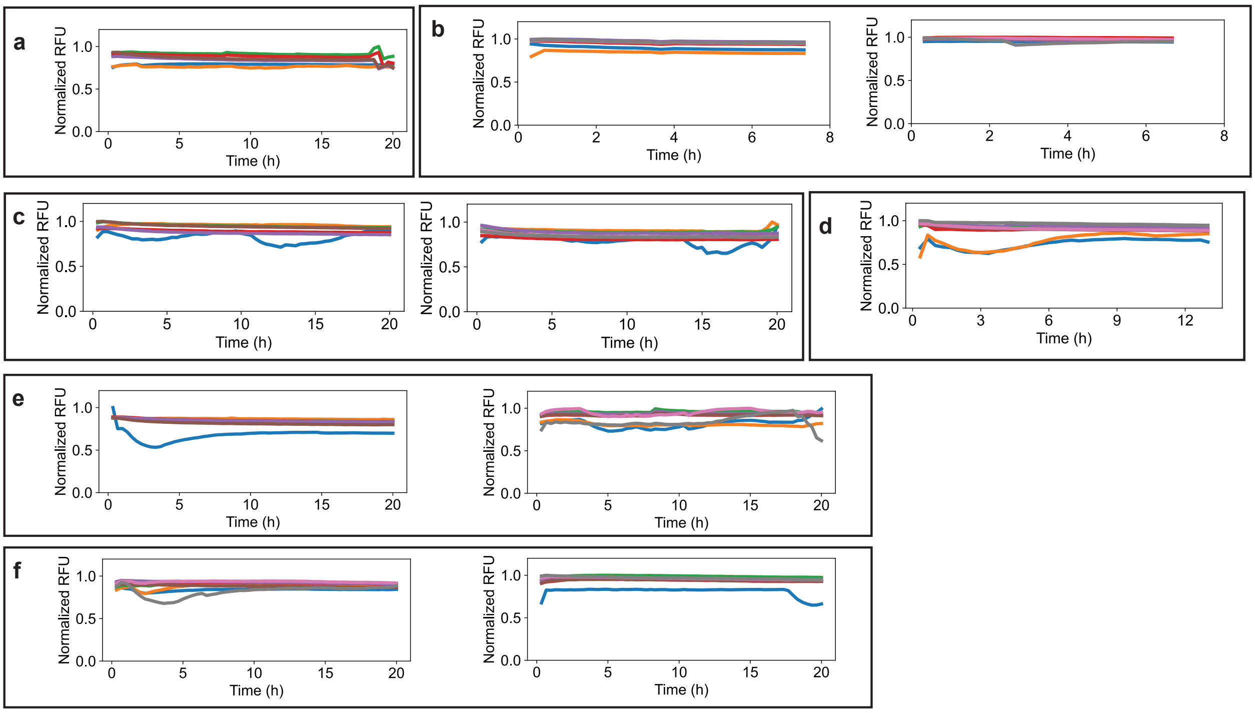

Supplementary Figure 6: **Fluorescence of mScarlet tracer for all the chemostat experiments discussed in this study.** **a,** Tracer signal for the experiment pertaining to Figure 4d. **b,** Tracer signal for the experiments pertaining to Figure 4e. **c,** Tracer signal for the experiments pertaining to Figure 4f. **d,** Tracer signal for the experiment pertaining to Figure 4g. **e,** Tracer signal for the experiments pertaining to Figure 4h. **f,** Tracer signal for the experiments pertaining to Figure 4i. The steady states observed in mScarlet tracer fluorescence for all rings validated fidelity of all experiments. Each line graph in the plots represents the mScarlet tracer in one ring over the course of an experiment. Source data are provided as a Source Data file.

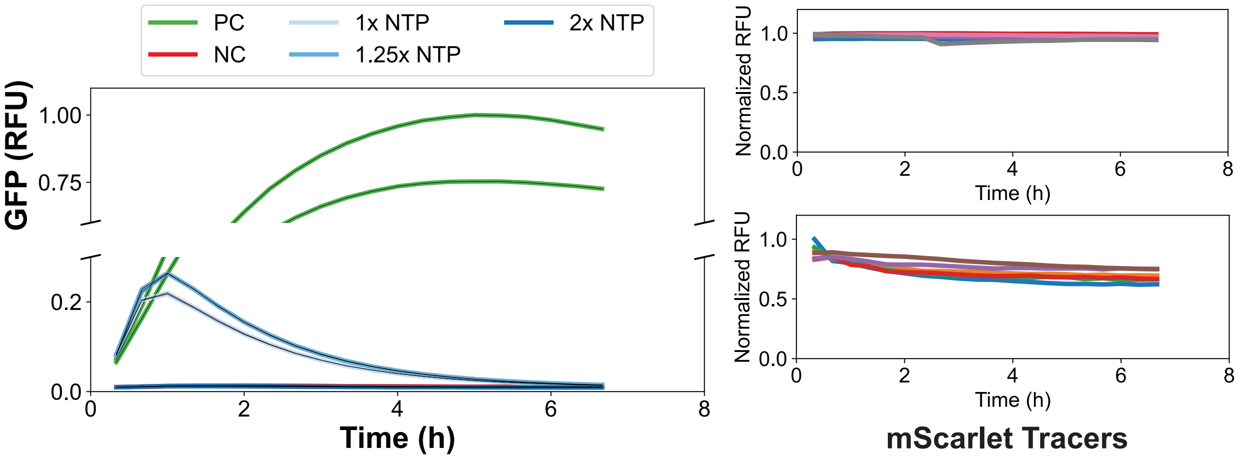

Supplementary Figure 7: **Titration of NTPs in chemostats**. The left panel shows the GFP fluorescence results on the chemostat on increasing the total concentration of NTPs to 1.25x and 2x its standard amount in a PURE reaction (n = 2 independent experiments). The graphs on the right track the tracer fluorescence for the relevant experiments, proving their validity. Each line graph in the plots represents the mScarlet tracer in one ring over the course of an experiment. Source data are provided as a Source Data file.

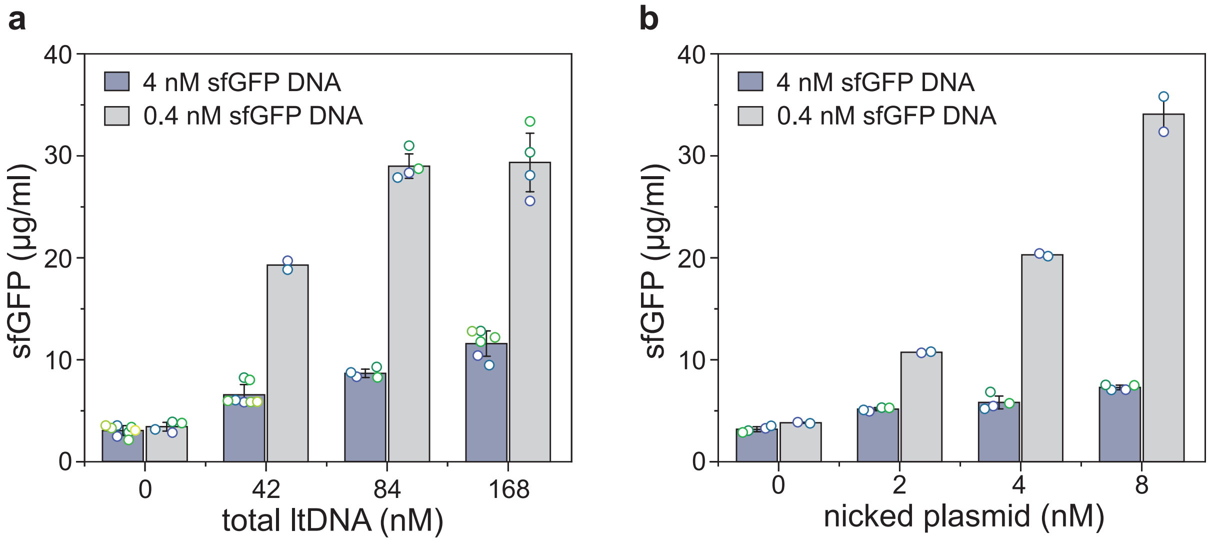

Supplementary Figure 8: **Titration of sfGFP template and tRNA templates.** **a,** sfGFP expression with 21 ltDNAs. With 4 nM sfGFP template, n = 8 independent experiments for 0 nM ltDNA, n = 7 for 42 nM ltDNA, n = 4 for 84 nM ltDNA, n = 6 for 168 nM ltDNA. With 0.4 nM sfGFP template, n = 4 for 0, 84 and 168 nM ltDNA, n = 2 for 42 nM ltDNA. **b**. sfGFP expression with nicked plasmid. With 4 nM sfGFP template, n = 4 for all tested nicked plasmid concentrations. With 0.4 nM sfGFP template, n = 2 for all tested nicked plasmid concentrations. Each dot represents a data point from an independent replicate. Bars and error bars represent the mean and standard deviation. Source data are provided as a Source Data file.

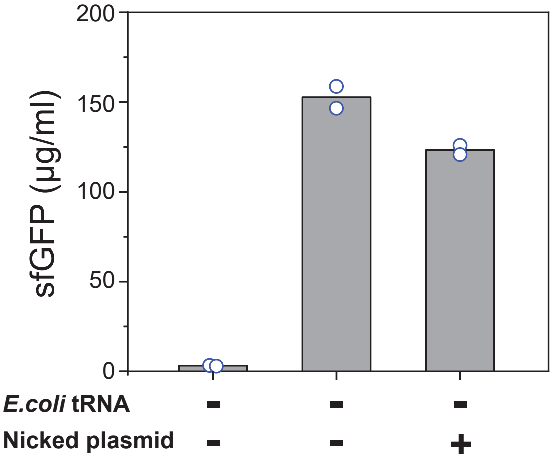

Supplementary Figure 9: **sfGFP expression in the presence of saturated concentration.** The addition of 8 nM nicked plasmid to reactions with 0.4 nM sfGFP template and saturated concentration of *E. coli* tRNA (4 μg/μL) reduced sfGFP yield (n = 2). Each dot represents a data point from an independent replicate. Bars represent the mean. Source data are provided as a Source Data file.

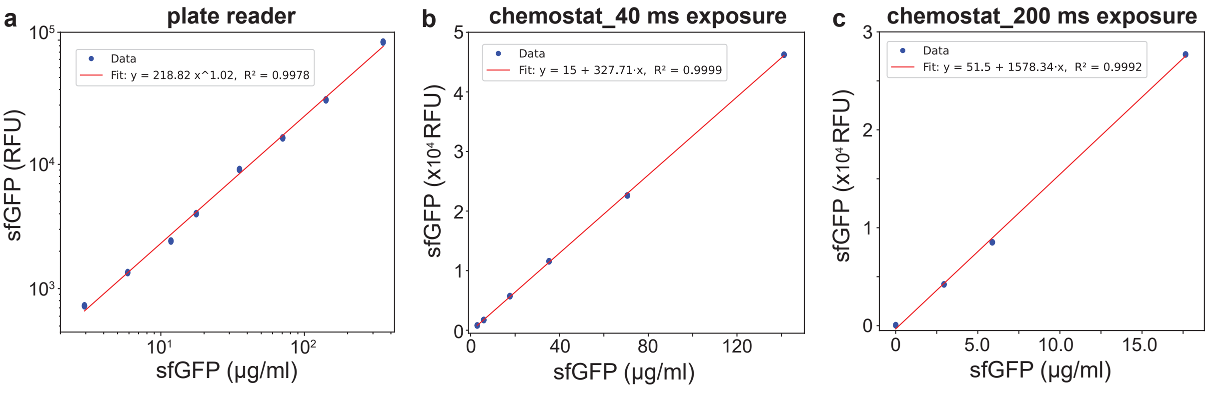

Supplementary Figure 10: **Calibration curves of sfGFP.** Calibration curves of sfGFP measured in plate reader (**a**) and in chemostat with 40 ms exposure (**b**) and 200 ms exposure (**c**). Source data are provided as a Source Data file.

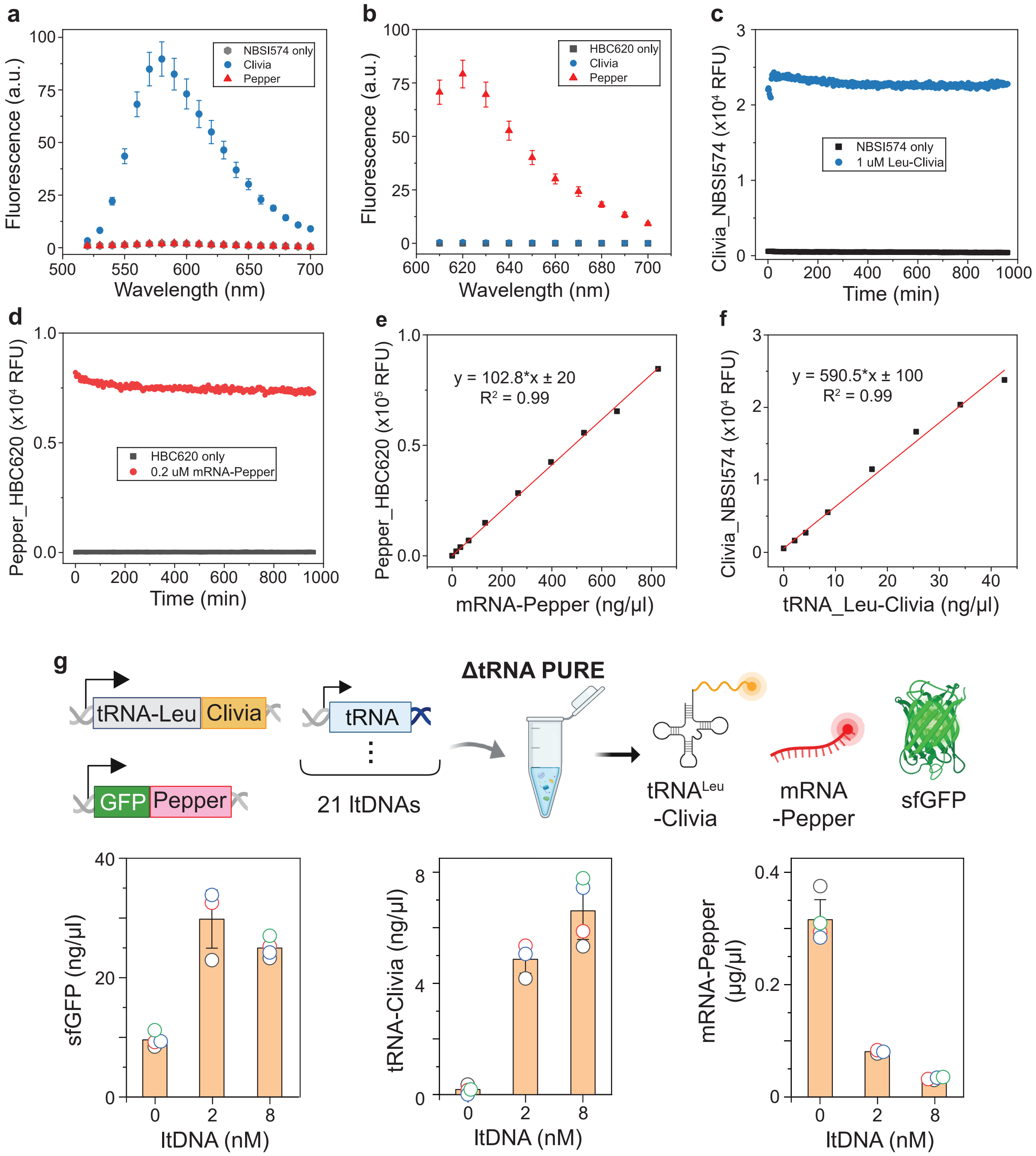

Supplementary Figure 11: Caption on next page

Supplementary Figure 11: **Aptamer assay.** **a**, Fluorescence spectra as measured upon 490 nm excitation of a solution containing 4 µM NBSI574 and 1 µM corresponding aptamer (mRNA-Pepper or tRNA-Clivia). **b**, Fluorescence spectra as measured upon 490 nm excitation of a solution containing 4 µM HBC620 and 1 µM corresponding aptamer (mRNA-Pepper or tRNA-Clivia). **c**, Stability of NBSI574 (3 µM) and $tRNA^{Leu}$-Clivia (1 µM) in buffer (40 mM HEPES pH 7.4, 125 mM KCl, 5 mM $MgCl_2$). **d**, Stability of HBC620 (3 µM) and mRNA-Pepper (0.2 µM) in buffer (40 mM HEPES pH 7.4, 125 mM KCl, 5 mM $MgCl_2$). **e-f**, Calibration curves for mRNA-Pepper (**e**) and $tRNA^{Leu}$-Clivia (**f**). **g**, Quantification of sfGFP, mRNA, and tRNA synthesis during ltDNA titration. The top panel shows the experimental design. The schematic was partially generated using BioRender. The genes for Pepper and Clivia apatmer were inserted after sfGFP and $tRNA^{Leu}$ genes respectively to quantify the mRNA and $tRNA^{Leu}$. The two templates were mixed with indicated concentration of 21 ltDNAs in the ΔtRNA PURE system. The concentration of $tRNA^{Leu}$-Clivia template was adjusted to the same level of individual ltDNA in each reaction, while 0.4 nM sfGFP-Pepper template was used for all reactions. The bottom panel shows fluorescence levels of sfGFP, mRNA-Pepper, and $tRNA^{Leu}$-Clivia as we titrated different concentrations of 21 ltDNAs to the system. n = 4 independent experiments with 0 and 8 nM ltDNA, n = 3 with 4 nM ltDNA. Each dot represents an independent replicate. Bars and error bars represent the mean and standard deviation. Source data are provided as a Source Data file.

Supplementary Table 1: A list of tRNA templates used in this study.

NO.	Name	Sequence (5'→3')
1	tRNA_Ala_GGC	CCGCGTAATACGACTCACTATAGGGGCTATAGCTCAGCTGGGAGAGCGCTTGCATGGCATGCAAGAGGTCAGCGGTTCGATCCCGCTTAGCTCCACCA
2	tRNA_Arg_CCG	CCGCGTAATACGACTCACTATAGCGCCCGTAGCTCAGCTGGATAGAGCGCTGCCCTCCGGAGGCAGAGGTCTCAGGTTCGAATCCTGTCGGGCGCGCCA
3	tRNA_Asn_GUU_mut	CCGCGTAATACGACTCACTATAGCCTCTGTAGTTCAGTCGGTAGAACGGCGGACTGTTAATCCGTATGTCACTGGTTCGAGTCCAGTCAGAGGCGCCA
4	tRNA_Asp_GUC	CCGCGTAATACGACTCACTATAGGAGCGGTAGTTCAGTCGGTTAGAATACCTGCCTGTCACGCAGGGGGTCGCGGGTTCGAGTCCCGTCCGTTCCGCCA
5	tRNA_Cys_GCA	CCGCGTAATACGACTCACTATAGGCGCGTTAACAAAGCGGTTATGTAGCGGATTGCAAATCCGTCTAGTCCGGTTCGACTCCGGAACGCGCCTCCA
6	tRNA_fMet_CAU_mut	CCGCGTAATACGACTCACTATAGGCGGGGTGGAGCAGCCTGGTAGCTCGTCGGGCTCATAACCCGAAGATCGTCGGTTCAAATCCGGCCCCCGCAACCA
7	tRNA_Gln_CUG	CCGCGTAATACGACTCACTATATGGGGTATCGCCAAGCGGTAAGGCACCGGATTCTGATTCCGGCATTCCGAGGTTCGAATCCTCGTACCCCAGCCA
8	tRNA_Glu_CUC	CCGCGTAATACGACTCACTATAGTCCCCTTCGTCTAGAGGCCCAGGACACCGCCCTCTCACGGCGGTAACAGGGGTTCGAATCCCCTAGGGGACGCCA
9	tRNA_Gly_GCC	CCGCGTAATACGACTCACTATAGCGGGAATAGCTCAGTTGGTAGAGCACGACCTTGCCAAGGTCGGGGTCGCGAGTTCGAGTCTCGTTTCCCGCTCCA
10	tRNA_His_GUG	CCGCGTAATACGACTCACTATAGGTGGCTATAGCTCAGTTGGTAGAGCCCTGGATTGTGATTCCAGTTGtCGTGGGTTCGAATCCCATTAGCCACCCCA
11	tRNA_Ile_GAU_ϕ2.5	CCGCGTAATACGACTCACTATTAGGCTTGTAGCTCAGGTGGTTAGAGCGCACCCCTGATAAGGGTGAGGTCGGTGGTTCAAGTCCACTCAGGCCTACCA
12	tRNA_Leu_CAG	CCGCGTAATACGACTCACTATAGCGAAGGTGGCGGAATTGGTAGACGCGCTAGCTTCAGGTGTTAGTGTCCTTACGGACGTGGGGGTTCAAGTCCCCCCCCTCGCACCA

Continued on next page...

NO.	Name	Sequence (5'→3')
13	tRNA_Lys_CUU	CCGCGTAATACGACTCACTATAGGGTCGTTAGCTCAGTTGGTAGAGCAGTTGAC TCTTAATCAATTGGTCGCAGGTTCGAATCCTGCACGACCCACCA
14	tRNA_mMet_CAU	CCGCGTAATACGACTCACTATAGGCTACGTAGCTCAGTTGGTTAGAGCACATCA CTCATAATGATGGGGTCACAGGTTCGAATCCCGTCGTAGCCACCA
15	tRNA_Phe_GAA	CCGCGTAATACGACTCACTATAGCCCGGATAGCTCAGTCGGTAGAGCAGGGGAT TGAAAATCCCCGTGTCCTTGGTTCGATTCCGAGTCCGGGCACCA
16	tRNA_Pro_GGG	CCGCGTAATACGACTCACTATACGGCACGTAGCGCAGCCTGGTAGCGCACCGTC ATGGGGTGTCGGGGGTCGGAGGTTCAAATCCTCTCGTGCCGACCA
17	tRNA_Ser_GGA	CCGCGTAATACGACTCACTATAGGTGAGGTGTCCGAGTGGCTGAAGGAGCACGC CTGGAAAGTGTGTATACGGCAACGTATCGGGGGTTCGAATCCCCCCCTCACCGC CA
18	tRNA_Thr_GGU	CCGCGTAATACGACTCACTATAGCTGATATGGCTCAGTTGGTAGAGCGCACCCT TGGTAAGGGTGAGGTCCCCAGTTCGACTCTGGGTATCAGCACCA
19	tRNA_Trp_CCA_ϕ2.5	CCGCGTAATACGACTCACTATTAGGGGCGTAGTTCAATTGGTAGAGCACCGGTC TCCAAAACCGGGTGtTGGGAGTTCGAGTCTCTCCGCCCCTGCCA
20	tRNA_Tyr_GUA	CCGCGTAATACGACTCACTATAGGTGGGGTTCCCGAGCGGCCAAAGGGAGCAGA CTGTAAATCTGCCGTCACAGACTTCGAAGGTTCGAATCCTTCCCCCACCACCA
21	tRNA_Val_GAC	CCGCGTAATACGACTCACTATAGCGTCCGTAGCTCAGTTGGTTAGAGCACCACC TTGACATGGTGGGGGTCGGTGGTTCGAGTCCACTCGGACGCACCA
22	tRNA_Asn_GUU	CCGCGTAATACGACTCACTATATCCTCTGTAGTTCAGTCGGTAGAACGGCGGAC TGTTAATCCGTATGTCACTGGTTCGAGTCCAGTCAGAGGAGCCA
23	tRNA_fMet_CAU	CCGCGTAATACGACTCACTATACGCGGGGTGGAGCAGCCTGGTAGCTCGTCGGG CTCATAACCCGAAGATCGTCGGTTCAAATCCGGCCCCCGCAACCA
24	tRNA_Ile_GAU	CCGCGTAATACGACTCACTATAAGGCTTGTAGCTCAGGTGGTTAGAGCGCACCC CTGATAAGGGTGAGGTCGGTGGTTCAAGTCCACTCAGGCCTACCA
25	tRNA_Trp_CCA	CCGCGTAATACGACTCACTATAAGGGGCGTAGTTCAATTGGTAGAGCACCGGTC TCCAAAACCGGGTGtTGGGAGTTCGAGTCTCTCCGCCCCTGCCA

Supplementary Table 2: A list of primers used in this study.

NO.	Name	Sequence (5'→3')
1	tRNA_Ala_F	CCGCGTAATACGACTCACTATAGGGGCTATAGCTCAGCTG
2	tRNA_Ala_R	TGGTGGAGCTAAGCGGGATCG
3	tRNA_Arg_F	CCGCGTAATACGACTCACTATAGCGCCCGTAGCTCAG
4	tRNA_Arg_R	TGGCGCGCCCGACAGGATTCG
5	tRNA_Asn_F	CCGCGTAATACGACTCACTATATCCTCTGTAGTTCAGTCG
6	tRNA_Asn_R	TGGCTCCTCTGACTGGACTCG
7	tRNA_Asn_mut_F	CCGCGTAATACGACTCACTATAGCCTCTGTAGTTCAGTCG
8	tRNA_Asn_mut_R	TGGCGCCTCTGACTGGACTCG
9	tRNA_Asp_F	CCGCGTAATACGACTCACTATAGGAGCGGTAGTTCAGTCG
10	tRNA_Asp_R	TGGCGGAACGGACGGGACTCG
11	tRNA_Cys_F	CCGCGTAATACGACTCACTATAGGCGCGTTAACAAAGCG
12	tRNA_Cys_R	TGGAGGCGCGTTCCGGAGTCG
13	tRNA_fMet_F	CCGCGTAATACGACTCACTATACGCGGGGTGGAGCAGCCTG
14	tRNA_fMet_R	TGGTTGCGGGGGCCGGA
15	tRNA_fMet_mut_F	CCGCGTAATACGACTCACTATAGGCGGGGTGGAGCAGCCTG
16	tRNA_Gln_F	CCGCGTAATACGACTCACTATATGGGGTATCGCCAAGCGG
17	tRNA_Gln_R	TGGCTGGGGTACGAGGATTCG
18	tRNA_Glu_F	CCGCGTAATACGACTCACTATAGTCCCCTTCGTCTAGAGG
19	tRNA_Glu_R	TGGCGTCCCCTAGGGGATTCG
20	tRNA_Gly_F	CCGCGTAATACGACTCACTATAGCGGGAATAGCTCAGTTG
21	tRNA_Gly_R	TGGAGCGGGAAACGAGAC
22	tRNA_His_F	CCGCGTAATACGACTCACTATAGGTGGCTATAGCTCAGTTG
23	tRNA_His_R	TGGGGTGGCTAATGGGATTCG
24	tRNA_Ile_F	CCGCGTAATACGACTCACTATAAGGCTTGTAGCTCAGGTG
25	tRNA_Ile_R	TGGTAGGCCTGAGTGGACTTG
26	tRNA_Ile_ϕ2.5_F	CAGTAATACGACTCACTATTAGGCTTGTAGCTCAGGTG

Continued on next page...

NO.	Name	Sequence (5'→3')
27	tRNA_Leu_F	CCGCGTAATACGACTCACTATAGCGAAGGTGGCGGAATTG
28	tRNA_Leu_R	TGGTGCGAGGGGGGGGA
29	tRNA_Lys_F	CCGCGTAATACGACTCACTATAGGGTCGTTAGCTCAGTTGG
30	tRNA_Lys_R	TGGTGGGTCGTGCAGGATT
31	tRNA_mMet_F	CCGCGTAATACGACTCACTATAGGCTACGTAGCTCAGTTG
32	tRNA_mMet_R	TGGTGGCTACGACGGGATTCG
33	tRNA_Phe_F	CCGCGTAATACGACTCACTATAGCCCGGATAGCTCAGTC
34	tRNA_Phe_R	TGGTGCCCGGACTCGGAA
35	tRNA_Pro_F	CCGCGTAATACGACTCACTATACGGCACGTAGCGCAGCCTG
36	tRNA_Pro_R	TGGTCGGCACGAGAGGATTT
37	tRNA_Ser_F	CCGCGTAATACGACTCACTATAGGTGAGGTGTCCGAGTG
38	tRNA_Ser_R	TGGCGGTGAGGGGGGGATTCG
39	tRNA_Thr_F	CCGCGTAATACGACTCACTATAGCTGATATGGCTCAGTTGG
40	tRNA_Thr_R	TGGTGCTGATACCCAGAGTCG
41	tRNA_Trp_F	CCGCGTAATACGACTCACTATAAGGGGCGTAGTTCAATTG
42	tRNA_Trp_R	TGGCAGGGGCGGAGAGACTCG
43	tRNA_Trp_ϕ2.5_F	CAGTAATACGACTCACTATTAGGGGCGTAGTTCAATTG
44	tRNA_Tyr_F	CCGCGTAATACGACTCACTATAGGTGGGGTTCCCGAG
45	tRNA_Tyr_R	TGGTGGTGGGGGAAGGATTCG
46	tRNA_Val_F	CCGCGTAATACGACTCACTATAGCGTCCGTAGCTCAGTTG
47	tRNA_Val_R	TGGTGCGTCCGAGTGGACTCG
48	T7F	CCGCGTAATACGACTCACTATA
49	T7R	AAAAAACCCCTCAAGACCCGTTTAGAGGC
50	Clivia_R	GGAAGTGTCTGCCTTTCGGCATGTTTAC
51	Pepper_R	TTGCCATGAATGATCCCGGCGCCAGTG

Supplementary Table 3: DNA template for protein and aptamer.

Name	Sequence (5'→3')
sfGFP	TAATACGACTCACTATAGGGGAATTGTGAGCGGATAACAATTCCCCTCTAGAAATAATTTTGTT<br>TAACTTTAAGAAGGAGATATACATATGTCTAAGGGTGAGGAGCTGTTTACTGGTGTTGTTCCTA<br>TTCTGGTTGAGCTGGACGGTGACGTTAACGGTCACAAGTTTTCTGTTCGGGGTGAGGGTGAGGG<br>TGACGCTACTAACGGTAAGCTGACTCTGAAGTTTATTTGTACTACTGGTAAGCTGCCTGTTCCT<br>TGGCCTACTCTGGTTACTACTCTGACTTACGGTGTTCAGTGTTTTTCTCGGTACCCTGACCACA<br>TGAAGCGGCACGACTTTTTTAAGTCTGCTATGCCTGAGGGTTACGTTCAGGAGCGGACTATTTC<br>TTTTAAGGACGACGGTACTTACAAGACTCGGGCTGAGGTTAAGTTTGAGGGTGACACTCTGGTT<br>AACCGGATTGAGCTGAAGGGTATTGACTTTAAGGAGGACGGTAACATTCTGGGTCACAAGCTGG<br>AGTACAACTTTAACTCTCACAACGTTTACATTACTGCTGACAAGCAGAAGAACGGTATTAAGGC<br>TAACTTTAAGATTCGGCACAACGTTGAGGACGGTTCTGTTCAGCTGGCTGACCACTACCAGCAG<br>AACACTCCTATTGGTGACGGTCCTGTTCTGCTGCCTGACAACCACTACCTGTCTACTCAGTCTG<br>TTCTGTCTAAGGACCCTAACGAGAAGCGGGACCACATGGTTCTGCTGGAGTTTGTTACTGCTGC<br>TGGTATTACTCACGGTATGGACGAGCTGTACAAGGGTTCTCACCACCACCACCACCACTAAGAT<br>CCGGCTGCTAACAAAGCCCGAAAGGAAGCTGAGTTGGCTGCTGCCACCGCTGAGCAATAACTAG<br>CATAACCCCTTGGGGCCTCTAAACGGGTCTTGAGGGGTTTTTT

Continued on next page...

Name	Sequence (5'→3')
sfGFP-Pepper	TAATACGACTCACTATAGGGAGACCACAACGGTTTCCCTCTAGAAATAATTTTGTTTAACTTTA<br>AGAAGGAGATATACCATGTCTAAGGGTGAGGAGCTGTTTACTGGTGTTGTTCCTATTCTGGTTG<br>AGCTGGACGGTGACGTTAACGGTCACAAGTTTTCTGTTCGGGGTGAGGGTGAGGGTGACGCTAC<br>TAACGGTAAGCTGACTCTGAAGTTTATTTGTACTACTGGTAAGCTGCCTGTTCCTTGGCCTACT<br>CTGGTTACTACTCTGACTTACGGTGTTCAGTGTTTTTCTCGGTACCCTGACCACATGAAGCGGC<br>ACGACTTTTTTAAGTCTGCTATGCCTGAGGGTTACGTTCAGGAGCGGACTATTTCTTTTAAGGA<br>CGACGGTACTTACAAGACTCGGGCTGAGGTTAAGTTTGAGGGTGACACTCTGGTTAACCGGATT<br>GAGCTGAAGGGTATTGACTTTAAGGAGGACGGTAACATTCTGGGTCACAAGCTGGAGTACAACT<br>TTAACTCTCACAACGTTTACATTACTGCTGACAAGCAGAAGAACGGTATTAAGGCTAACTTTAA<br>GATTCGGCACAACGTTGAGGACGGTTCTGTTCAGCTGGCTGACCACTACCAGCAGAACACTCCT<br>ATTGGTGACGGTCCTGTTCTGCTGCCTGACAACCACTACCTGTCTACTCAGTCTGTTCTGTCTA<br>AGGACCCTAACGAGAAGCGGGACCACATGGTTCTGCTGGAGTTTGTTACTGCTGCTGGTATTAC<br>TCACGGTATGGACGAGCTGTACAAGGGTTCTCACCACCACCACCACCACTAAGGCAGCTAAAGG<br>GTGATCTTGCCATGTGTATGTGGGTTCGCCCACATACTCTGATGATCCCCAATCGTGGCGTGTC<br>GGCCTGCTTCGGCAGGCACTGGCGCCGGGATCATTCATGGCAACGGCTGCTAACAAAGCCCGAA<br>AGGAAGCTGAGTTGGCTGCTGCCACCGCTGAGCAATAACTAGCATAACCCCTTGGGGCCTCTAA<br>ACGGGTCTTGAGGGGTTTTTTGCTGAAAGGAGGAACTATATCC

Continued on next page...

Name	Sequence (5'→3')
mCherry-Broccoli	CCGCGTAATACGACTCACTATAGGGAGACCACAACGGTTTCCCTCTAGAAATAATTTTGTTTAA<br>CTTTAAGAAGGAGATATACCATGTCTGGTTCTCACCACCACCACCACCACGGTTCTTCTGGTGA<br>GAACCTGTACTTTCAGTCTATGGTTTCTAAGGGTGAGGAGGACAACATGGCTATTATTAAGGAG<br>TTTATGCGGTTTAAGGTTCACATGGAGGGTTCTGTTAACGGTCACGAGTTTGAGATTGAGGGTG<br>AGGGTGAGGGTCGGCCTTACGAGGGTACTCAGACTGCTAAGCTGAAGGTTACTAAGGGTGGTCC<br>TCTGCCTTTTGCTTGGGACATTCTGTCTCCTCAGTTTATGTACGGTTCTAAGGCTTACGTTAAG<br>CACCCTGCTGACATTCCTGACTACCTGAAGCTGTCTTTTCCTGAGGGTTTTAAGTGGGAGCGGG<br>TTATGAACTTTGAGGACGGTGGTGTTGTTACTGTTACTCAGGACTCTTCTCTGCAGGACGGTGA<br>GTTTATTTACAAGGTTAAGCTGCGGGGTACTAACTTTCCTTCTGACGGTCCTGTTATGCAGAAG<br>AAGACTATGGGTTGGGAGGCTTCTTCTGAGCGGATGTACCCTGAGGACGGTGCTCTGAAGGGTG<br>AGATTAAGCAGCGGCTGAAGCTGAAGGACGGTGGTCACTACGACGCTGAGGTTAAGACTACTTA<br>CAAGGCTAAGAAGCCTGTTCAGCTGCCTGGTGCTTACAACGTTAACATTAAGCTGGACATTACT<br>TCTCACAACGAGGACTACACTATTGTTGAGCAGTACGAGCGGGCTGAGGGTCGGCACTCTACTG<br>GTGGTATGGACGAGCTGTACAAGTAAAGGGTGATCTTGCCATGTGTATGTGGGAGACGGTCGGG<br>TCCAGATATTCGTATCTGTCGAGTAGAGTGTGGGCTCCCACATACTCTGATGATCCTTCGGGAT<br>CATTCATGGCAACGGCTGCTAACAAAGCCCGAAAGGAAGCTGAGTTGGCTGCTGCCACCGCTGA<br>GCAATAACTAGCATAACCCCTTGGGGCCTCTAAACGGGTCTTGAGGGGTTTTTTGCTGAAAGGA<br>GGAACTATATCC
tRNA^{Leu}-Clivia	CCGCGTAATACGACTCACTATAGCGAAGGTGGCGGAATTGGTAGACGCGCTAGCTTCAGGTGTT<br>AGTGTCCTTACGGACGTGGGGGTTCAAGTCCCCCCCCTCGCACCAAGTCCTATGTTGCCATGTG<br>TATGTGGGTTCGCCCACATACTCTGATGATCCCCAATCGTGGCGTGTCGGCCTGCTTCGGCAGG<br>CACTGGCGCCGGGATCATTCATGGCAA

Continued on next page...

**Name**	**Sequence (5'→3')**
T.m tRNase Z	ATGCATCATCATCATCATCATAGCGGCATGAACATTATTGGCTTTAGCAAAGCGCTGTTTAGCA<br>CCTGGATTTATTATAGCCCGGAACGCATTCTGTTTGATGCGGGCGAAGGCGTGAGCACCACCCT<br>GGGCAGCAAAGTGTATGCGTTTAAATATGTGTTTCTGACCCATGGCCATGTGGATCATATTGCG<br>GGCCTGTGGGGCGTGGTGAACATTCGCAACAACGGCATGGGCGATCGCGAAAAACCGCTGGATG<br>TGTTTTATCCGGAAGGCAACCGCGCGGTGGAAGAATATACCGAATTTATTAAACGCGCGAACCC<br>GGATCTGCGCTTTAGCTTTAACGTGCATCCGCTGAAAGAAGGCGAACGCGTGTTTCTGCGCAAC<br>GCGGGCGGCTTTAAACGCTATGTGCAGCCGTTTCGCACCAAACATGTGAGCAGCGAAGTGAGCT<br>TTGGCTATCATATTTTTGAAGTGCGCCGCAAACTGAAAAAAGAATTTCAGGGCCTGGATAGCAA<br>AGAAATTAGCCGCCTGGTGAAAGAAAAAGGCCGCGATTTTGTGACCGAAGAATATCATAAAAAA<br>GTGCTGACCATTAGCGGCGATAGCCTGGCGCTGGATCCGGAAGAAATTCGCGGCACCGAACTGC<br>TGATTCATGAATGCACCTTTCTGGATGCGCGCGATCGCCGCTATAAAAACCATGCGGCGATTGA<br>TGAAGTGATGGAAAGCGTGAAAGCGGCGGGCGTGAAAAAAGTGATTCTGTATCATATTAGCACC<br>CGCTATATTCGCCAGCTGAAAAGCGTGATTAAAAAATATCGCGAAGAAATGCCGGATGTGGAAA<br>TTCTGTATATGGATCCGCGCAAAGTGTTTGAAATGTAA

Supplementary Table 4: A reduced codon table [1] used for protein template design.

NO.	Amino acid	Codon (5' $\rightarrow$ 3')	Anticodon (5' $\rightarrow$ 3')
1	Ala	GCU	GGC
2	Arg	CGG	CCG
3	Asn	AAC	GUU
4	Asp	GAC	GUC
5	Cys	UGU	GCA
6	Gln	CAG	CUG
7	Glu	GAG	CUC
8	Gly	GGU	GCC
9	His	CAC	GCG
10	Ile	AUU	GAU
11	Leu	CUG	CAG
12	Lys	AAG	CUU
13	Met	AUG	CAU
14	Phe	UUU	GAA
15	Pro	CCU	GGG
16	Ser	UCU	GGA
17	Thr	ACU	GGU
18	Trp	UGG	CCA
19	Tyr	UAC	GUA
20	Val	GUU	GAC
21	Stop codon	UAA	

Supplementary Table 5: Chemostat initialization protocol

**Initial fill**			
Step	Operation	Solution	Ring number
Initialize reactions in all rings			
0B	Addition of protein/ribosome PURE mix		
	Flush rings	Buffer	1-8
	Flush rings	Protein/ribosome PURE mix	1-8
0C	Addition of DNA-Energy mix		
	Load 60%	Positive control DNA-Energy mix	1-2
	Load 60%	DNA-Energy mix w/ tRNA templates 1	3-4
	Load 60%	DNA-Energy mix w/ tRNA templates 2	5-6
	Load 60%	Negative control DNA-Energy mix	7-8
0D	Incubation with continuous mixing		
Dilutions of 20% every 20 minutes			

Supplementary Table 6: Dilution protocol for long-term chemostat experiments

**Self-regeneration**			
Step	Operation	Solution	Ring number
Imaging and dilution step every 20 minutes			
1A	Image each reactor ring		
1B	Addition of protein/ribosome PURE mix		
	Load 20%	Buffer	1-8
	Load 20%	Protein/ribosome PURE mix	1-8
1C	Addition of DNA-Energy mix		
	Load 12%	Buffer	1-8
	Load 12%	Positive control DNA-Energy mix	1-2
	Load 12%	DNA-Energy mix w/ tRNA templates 1	3-4
	Load 12%	DNA-Energy mix w/ tRNA templates 2	5-6
	Load 12%	Negative control DNA-Energy mix	7-8
1D	Incubation with continuous mixing		
Mixing for 20 minutes			

References

[1] Keita Hibi, Kazuaki Amikura, Naoki Sugiura, Keiko Masuda, Satoshi Ohno, Takashi Yokogawa, Takuya Ueda, and Yoshihiro Shimizu. Reconstituted cell-free protein synthesis using in vitro transcribed trnas. *Communications Biology*, 3:350, 2020.
